# The Utility of Language Models in Cardiology: A Narrative Review of the Benefits and Concerns of ChatGPT-4

**DOI:** 10.3390/ijerph20156438

**Published:** 2023-07-25

**Authors:** Dhir Gala, Amgad N. Makaryus

**Affiliations:** 1 Department of Clinical Science, American University of the Caribbean School of Medicine, Cupecoy, Sint Maarten, The Netherlands; dhirgala@gmail.com; 2 Donald and Barbara Zucker School of Medicine at Hofstra/Northwell, Hofstra University, 500 Hofstra Blvd., Hempstead, NY 11549, USA; 3 Department of Cardiology, Nassau University Medical Center, Hempstead, NY 11554, USA

**Keywords:** language models, artificial intelligence, ChatGPT, generative artificial intelligence, generative language models, cardiology, medical education, patient outcomes

## Abstract

Artificial intelligence (AI) and language models such as ChatGPT-4 (Generative Pretrained Transformer) have made tremendous advances recently and are rapidly transforming the landscape of medicine. Cardiology is among many of the specialties that utilize AI with the intention of improving patient care. Generative AI, with the use of its advanced machine learning algorithms, has the potential to diagnose heart disease and recommend management options suitable for the patient. This may lead to improved patient outcomes not only by recommending the best treatment plan but also by increasing physician efficiency. Language models could assist physicians with administrative tasks, allowing them to spend more time on patient care. However, there are several concerns with the use of AI and language models in the field of medicine. These technologies may not be the most up-to-date with the latest research and could provide outdated information, which may lead to an adverse event. Secondly, AI tools can be expensive, leading to increased healthcare costs and reduced accessibility to the general population. There is also concern about the loss of the human touch and empathy as AI becomes more mainstream. Healthcare professionals would need to be adequately trained to utilize these tools. While AI and language models have many beneficial traits, all healthcare providers need to be involved and aware of generative AI so as to assure its optimal use and mitigate any potential risks and challenges associated with its implementation. In this review, we discuss the various uses of language models in the field of cardiology.

## 1. Introduction

Artificial intelligence (AI) is changing the way medicine is practiced. AI refers to the capability of machines to do tasks that require human intelligence, such as recognizing speech, making decisions, and learning [1]. This is accomplished through the use of algorithms, computer programs that can process data and make predictions based on patterns in that data. One of the most intriguing applications of AI in natural language processing has resulted in the creation of complex language models [2]. Language models are AI systems that use machine learning algorithms to comprehend and produce text like a human. Their sophisticated design enables them to create logical and contextually appropriate text by learning language structure and patterns from vast amounts of text data that is used to train the language models [3]. 

Language models are created using a process called machine learning, which involves training a computer program to recognize patterns in data. In the case of language models, the program is trained on a large dataset of text, such as books, online articles, or social media posts. The program uses sophisticated statistical methods to identify patterns in the language, such as the probability of certain words appearing together or the likelihood of a particular sentence structure. Once the program is trained, it can be used for a variety of tasks. One common use of language models is in natural language processing (NLP), which involves analyzing and understanding human language [4]. For example, a language model can be used to automatically summarize a long text document or to extract key information from a customer support chat conversation. Another use of language models is in speech recognition, where they are used to convert spoken language into text [5]. This is particularly useful for virtual assistants like Siri or Alexa, which rely on language models to understand and respond to user requests. Language models can also be used for machine translation, where they are used to automatically translate text from one language to another [6]. This is a particularly challenging task, as languages have different grammatical structures and idioms that can be difficult to translate accurately. However, recent advances in language models have led to significant improvements in machine translation accuracy. Finally, language models are increasingly being used in research to analyze text data and to generate new insights [7,8].

A number of uses for language models exist, such as chatbots, sentiment analysis, and language translation [9]. One of the most well-known language models is OpenAI’s ChatGPT-4 (Generative Pre-trained Transformer 4), which is capable of writing various texts such as poetry, essays, and even computer code [10]. BERT (Bidirectional Encoder Representations from Transformers) is another language model created by Google and is capable of comprehending the context and meaning of words in a sentence [11].

Language models have recently entered the medical field and have the potential to completely change how healthcare providers communicate with patients. Language models can be used to automate administrative tasks, enhance patient outcomes, and even help with medical diagnosis [2]. Medical records and other types of healthcare data can be analyzed using language models, which can reveal information about patient outcomes and available treatments. Accurately diagnosing and foreseeing the progression of cardiovascular diseases (CVDs) is one of cardiology’s biggest challenges. To gain insights into the diagnosis and treatment of CVDs, AI-powered language models have the capacity to analyze vast amounts of patient data, including medical records, test results, and clinical observations. This review aims to identify the various uses of language models in the field of cardiology.

## 2. Language Models in Cardiology

### 2.1. Medical Diagnosis and Decision Support

AI and language models have the potential to revolutionize medical diagnosis and decision support in cardiology by analyzing vast amounts of medical data and assisting healthcare professionals in making more accurate diagnoses and treatment decisions. Analyzing medical images like echocardiograms, angiograms, and electrocardiograms (ECGs) is one way that AI and language models can assist in cardiology. It is possible to train AI algorithms to recognize patterns in these images that may be challenging for human clinicians. AI can be used, for instance, to identify abnormalities in ECGs that may be a sign of heart failure, ischemic heart disease, or arrhythmias [12].

**Figure 1:**
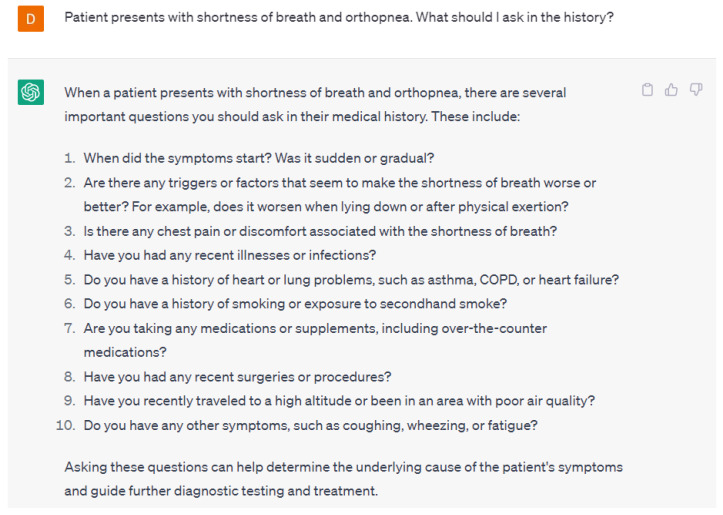
A response from ChatGPT-4 for the question “Patient presents with shortness of breath and orthopnea. What should I ask in the history?” [15].

**Figure 2:**
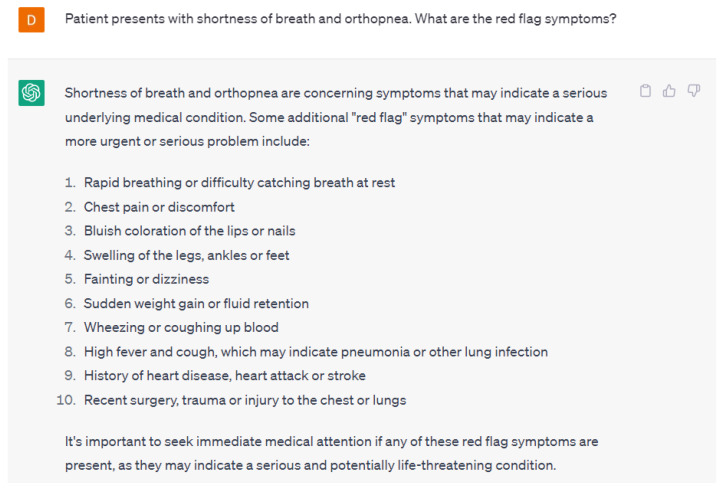
A response from ChatGPT-4 for the question “Patient presents with shortness of breath and orthopnea. What are some red flag symptoms?” [15].

Language models can also help in cardiology by analyzing patient medical records and other relevant data, such as family history and lifestyle factors [13,14]. Language models can identify patterns in this data that may be indicative of certain cardiovascular conditions or risk factors, enabling clinicians to make more accurate diagnoses and treatment recommendations. For example, language models can analyze patient data to predict the risk of developing cardiovascular disease and recommend appropriate lifestyle modifications or medications to reduce that risk. They can aid in early diagnosis and treatment by identifying patients who are most at risk of contracting specific cardiac diseases. Language models can guide healthcare providers on the appropriate questions to ask patients. Figure 1 gives an example of the follow-up questions necessary to appropriately diagnose a patient presenting with symptoms of shortness of breath and orthopnea from ChatGPT-4. Secondly, language models can assist in identifying potential red-flag symptoms that may require urgent treatment. ChatGPT-4 was able to appropriately identify potential red flag symptoms in patients presenting with shortness of breath and orthopnea (Figure 2). The responses provided by ChatGPT-4 are appropriate starting points for healthcare providers.

In addition to diagnosis, AI and language models can also assist in decision support by analyzing medical data and providing personalized treatment recommendations in cardiology. For example, AI can be used to analyze patient data and identify the most effective treatment options for a particular cardiovascular condition, based on factors such as the patient’s age, medical history, and genetic profile. AI can also help monitor patients with implanted devices such as pacemakers or defibrillators and alert clinicians to potential issues or malfunctions.

**Figure 3:**
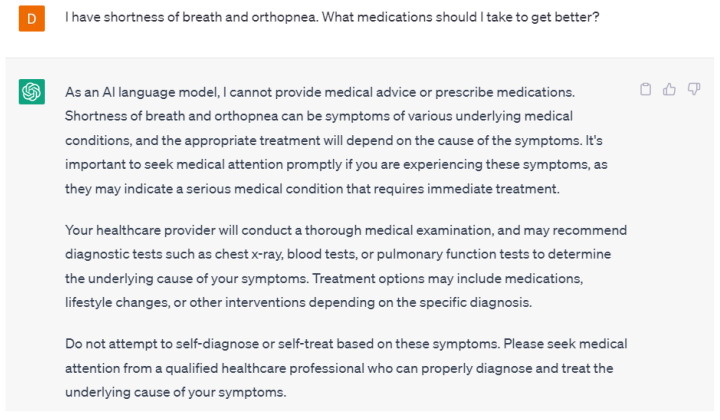
A response from ChatGPT-4 for the question “I have shortness of breath and orthopnea. What medications should I take to get better?” [15].

**Figure 4:**
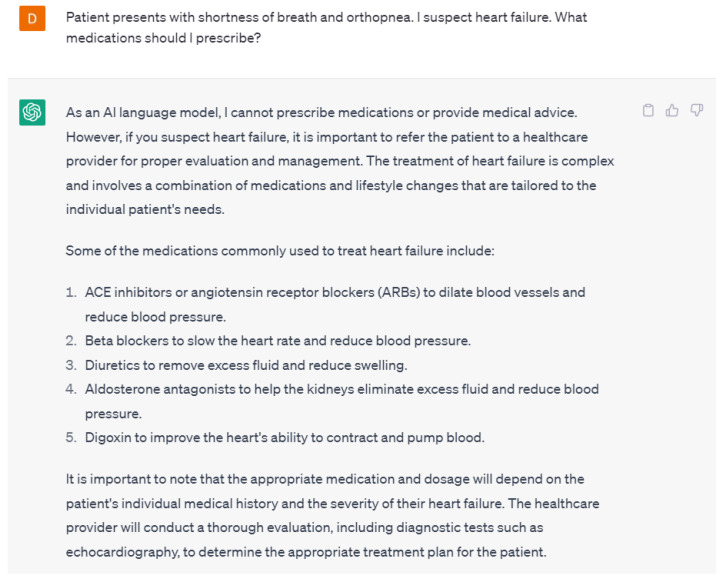
A response from ChatGPT-4 for the question “Patient presents with shortness of breath and orthopnea. I suspect heart failure. What medications should I prescribe?” [15].

A potential concern about language models is patients self-diagnosing and treating their symptoms. Interestingly, ChatGPT-4 did not suggest any medications or treatments when asked about it but instead recommended visiting a healthcare provider (Figure 3). From a healthcare provider’s perspective, ChatGPT-4 was able to suggest medications used to treat heart failure but did not provide a specific medication or prescription (Figure 4). These responses from ChatGPT-4 are appropriate as there is always a concern about patients self-treating their symptoms or conditions without consulting healthcare providers. From the healthcare provider’s perspective, although the responses were correct, they were not the gold-standard goal-directed medical therapy. This could have been due to the lack of training of ChatGPT-4 on the various guidelines or the lack of a specific prompt. It is crucial to make sure that the training data used to create language models is not only correct but also up-to-date to provide the most accurate response.

Language models can guide healthcare providers on the appropriate questions to ask patients during consultations, in addition to identifying at-risk patients. Language models can help healthcare providers narrow down potential diagnoses and suggest appropriate treatment plans by analyzing the patient’s symptoms, medical history, and other relevant information.

Despite these benefits, there are some important considerations to keep in mind while utilizing language models. The language models are limited to the quality and quantity of data used to train them, which may limit their ability to make accurate predictions or recommendations. Secondly, as language models rely only on information provided, they may be biased and fail to account for all relevant factors that may influence a patient’s diagnosis or treatment plan. Lastly, language models may be incapable of accounting for the nuances and complexities of individual patient cases especially rare pathologies or uncommon presentations, leading to incorrect recommendations or diagnoses.

### 2.2. Patient Communication and Education

AI and language models can also help in patient communication and education in the field of cardiology. By analyzing patient data, these technologies can provide personalized recommendations to patients, help them understand their medical conditions, and improve their adherence to treatment plans.

**Figure 5:**
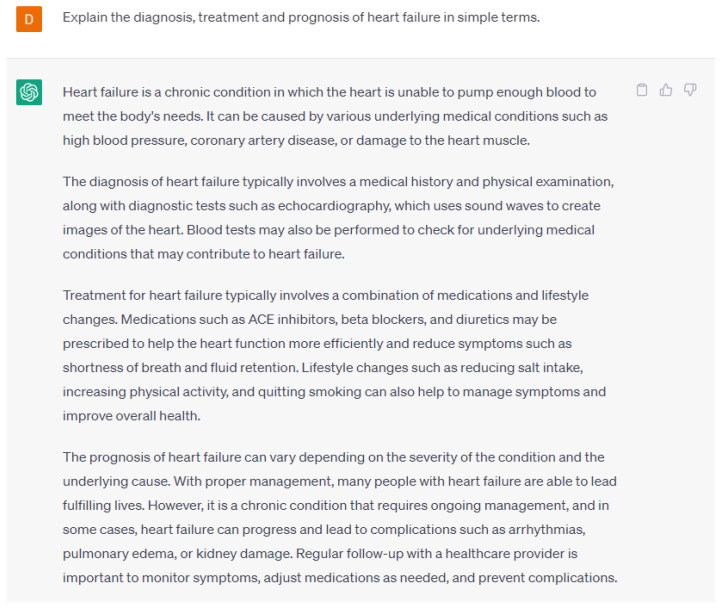
A response from ChatGPT-4 for the question “Explain the diagnosis, treatment, and prognosis of heart failure in simple terms” [15].

One way that AI and language models can help in patient communication and education in cardiology is by providing tailored education materials for patients [16,17]. For example, a language model could analyze a patient’s medical records and generate personalized educational content that explains their specific condition, treatment options, and potential risks and benefits. This can help patients better understand their diagnosis and treatment, which may lead to increased adherence to treatment plans and improved outcomes. By using language models, it is possible to produce patient education materials that are easy to comprehend and accessible to a range of patients. They can be used to generate individualized health recommendations and provide patients with real-time feedback on their health based on patient data. An example of educational material about heart failure is seen in Figure 5. These responses can be tailored to patients’ specific education levels, interests, and prognoses. By utilizing language models, healthcare providers can provide patients with appropriate personalized explanations and written materials compared to utilizing a general text for all patients. 

AI can also be used to develop chatbots or other conversational interfaces that can interact with patients and answer their questions in real time. For example, a patient with a heart condition could interact with a chatbot that provides information about their specific condition, medication, and lifestyle recommendations. The chatbot could also offer reminders for medications or appointments, which could help patients adhere to their treatment plans and manage their condition more effectively.

In addition, AI and language models can analyze patient data to identify patterns and provide proactive recommendations for lifestyle modifications [18]. For example, a language model could analyze a patient’s medical records and lifestyle data to identify factors that contribute to their cardiovascular disease risk, such as smoking, obesity, or lack of exercise. The model could then provide personalized recommendations for diet and exercise modifications, which could help patients improve their cardiovascular health.

There are some pitfalls that healthcare providers need to understand when using language models. There is a chance that language models may provide inaccurate or irrelevant information which can lead to misunderstanding or misinterpretation of medical information. Language models may also struggle to provide information in different languages, and these will need to be verified by a medical translator before providing it to the patient. Lastly, language models may also be limited by their inability to account for an individual patient’s unique needs and preferences. It is essential that any material generated through the use of AI be reviewed by an appropriately credentialed provider to assure accuracy.

### 2.3. Medical Education and Training

AI and language models can also help in medical education and training in the field of cardiology. These technologies can analyze large amounts of medical data and provide insights and recommendations to healthcare professionals, enabling them to make more accurate diagnoses and treatment decisions. One way that AI and language models can help in medical education and training in cardiology is by providing access to educational materials and resources. For example, a language model could analyze medical textbooks, research papers, and other resources to provide healthcare professionals with the latest information about cardiovascular conditions and treatment options. This could help clinicians stay up-to-date on the latest advances in cardiology and provide the best possible care to their patients.

**Figure 6:**
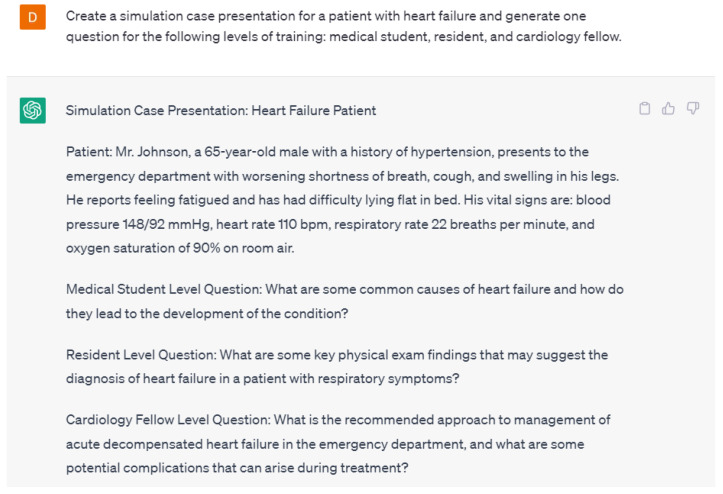
A response from ChatGPT-4 for the question “Create a simulation case presentation for a patient with heart failure and generate one question for the following levels of training: medical student, resident, and cardiology fellow” [15].

**Figure 7:**
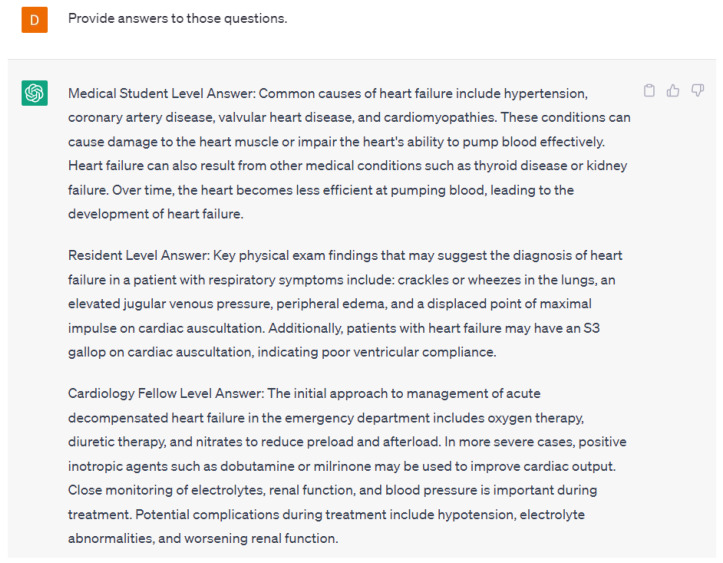
A response from ChatGPT-4 for the question “Provide answers to those questions” [15].

AI can also be used to develop virtual training tools and simulations for healthcare professionals. For example, a virtual training tool could simulate a patient with a cardiac condition and allow healthcare professionals to practice diagnosing and treating the condition in a realistic setting. This could help clinicians develop their skills and improve their confidence in managing cardiovascular conditions. Language models can be used to create educational materials for medical students and healthcare professionals. By simulating real-world clinical scenarios, they can help train medical professionals to make more accurate diagnoses and treatment decisions. Language models are able to create simulated cases and questions for varying levels of training in the healthcare field. An example case created by ChatGPT-4 is shown in Figure 6. Additionally, ChatGPT-4 was able to generate three questions about the case for different levels of training such as medical students, residents, and cardiology fellows. ChatGPT-4 also provided answers for all of those questions (Figure 7). The case and the follow-up questions generated by ChatGPT-4 were appropriate in terms of context and the various levels of training.

In addition, AI and language models can analyze patient data to identify patterns and provide insights into the most effective treatment options for specific cardiovascular conditions. This could help healthcare professionals develop more effective treatment plans and improve patient outcomes. For example, a language model could analyze patient data to identify the most effective medications and dosages for a patient with heart failure.

There are limitations to the use of language models in education. Language models may not be able to provide the same level of hands-on experience and exposure to real-world clinical scenarios compared to traditional teaching models. Language models may also have difficulty accounting for cultural or contextual factors that may influence medical practice and patient outcomes.

### 2.4. Streamlining Administrative Tasks

AI and language models can also help streamline administrative tasks in the field of cardiology. By automating routine administrative tasks, these technologies can free up time for healthcare professionals to focus on patient care and improve efficiency in healthcare operations.

One way that AI and language models can help streamline administrative tasks in cardiology is by automating documentation and record-keeping. For example, a language model could be used to automatically transcribe physician notes and patient medical records, reducing the need for manual data entry and allowing healthcare professionals to spend more time with their patients. This could also improve accuracy and reduce the risk of errors in patient records.

**Figure 8:**
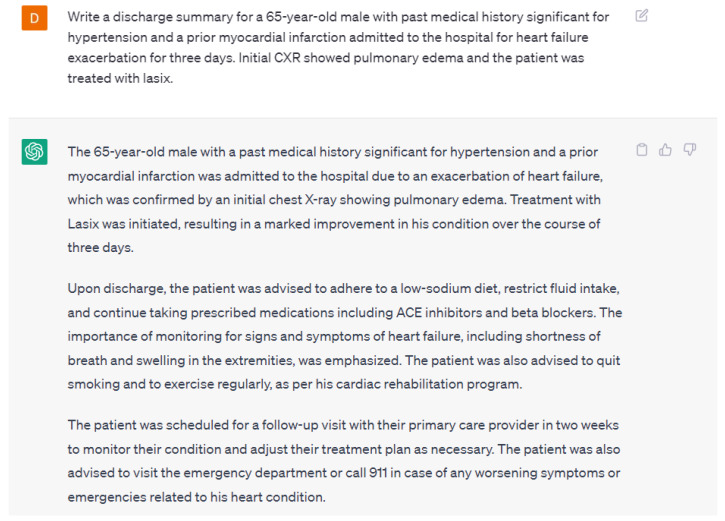
A response from ChatGPT-4 for the question “Write a discharge summary for a 65-year-old male with a past medical history significant for hypertension and a prior myocardial infarction admitted to the hospital for heart failure exacerbation for three days. Initial CXR showed pulmonary edema and the patient was treated with Lasix” [15].

Generative AI will be able to offload many medical provider tasks that have robbed healthcare providers of their central role of interacting with our patients on a humanistic level [19]. The never-ending task of composition and completion of medical records for appropriate documentation is an essential endeavor and will be augmented and improved by utilizing AI. AI can be utilized to update progress notes, summarize the results of various tests, and write discharge summaries. An example discharge summary written by ChatGPT-4 is in Figure 8. Although, the discharge summary was appropriate, ChatGPT-4 added additional information that was not provided in the input. This may lead to misleading information and healthcare providers need to be aware of these flaws. This can potentially be prevented by providing in-depth input that includes all the essential information and training ChatGPT-4 to avoid adding additional information. 

Additionally, other regulatory and required tasks such as obtaining pre-approvals and filing appeals for medical care will be made easier for providers by utilizing generative AI. The automation of these tasks will allow physicians to spend more time caring for patients and improving patient outcomes. 

Another great area of benefit will be in the gathering, synthesis, and summarization of ever-increasing amounts of data for each patient to make sure nothing is missed in the consideration of the differential diagnosis [20,21]. By freeing up providers from these and many more time-consuming and low-value tasks, care providers will be able to focus more attention on patient interaction through the ideal gathering of an appropriate history and performance of a complete physical exam. By focusing on this, maintenance of the emotional intelligence central to the humanistic approach in medicine is assured. Additionally, assuring a collaborative healthcare environment for interprofessional teams requires optimal communication, role clarification, and optimal relationships between team members [22,23]. These qualities require time to develop.

AI can also be used to develop predictive analytics tools that can help healthcare organizations better manage patient populations. For example, a predictive analytics tool could analyze patient data and identify those who are at high risk of developing cardiovascular disease. Healthcare organizations can then proactively reach out to these patients with preventative care measures, such as lifestyle recommendations and early interventions, before their conditions worsen. In addition, AI and language models can help improve communication and collaboration between healthcare professionals. For example, a language model could be used to automatically schedule appointments, coordinate patient care between different healthcare providers, and manage electronic health records. This could improve efficiency and reduce the risk of miscommunication between healthcare professionals.

## 3. Discussion

ChatGPT-4, especially when used with chatbots, is the perfect tool for human-machine conversations because of its ability to generate meaningful text. In this manuscript, we demonstrate how ChatGPT-4 can be used within the field of cardiology [15]. The use of language models and AI in medicine has the potential to enhance physician productivity and improve patient outcomes. The diagnosis and management of various cardiovascular diseases could be aided by AI and language models in the field of cardiology. To gain insights into the diagnosis and treatment of conditions, language models can analyze enormous amounts of patient data, including medical records, test results, and clinical observations [24]. By identifying patients who are most likely to develop particular cardiac diseases, language models can help medical professionals in the early diagnosis and treatment of cardiac conditions [25,26]. They can assist in identifying potential red flag symptoms that may require urgent treatment and instruct healthcare professionals on the right questions to ask patients during consultations.

In cardiology, the use of language models can help with medical diagnosis and decision-making [27,28]. By examining the patient’s symptoms, medical history, and other pertinent data, they can aid healthcare professionals in reducing the number of potential diagnoses and making suitable treatment recommendations. AI can also assure that the diagnostic imaging performed on cardiology patients is scrutinized and evaluated appropriately [29]. Additionally, language models can be used to create patient education materials that are simple to understand and useful for a variety of patients.

Although AI and language models have several benefits in clinical practice, it is important to acknowledge and address the limitations of their use. One concern is that these technologies might not always reflect the most up-to-date scientific information and may offer out-of-date information. Secondly, the cost of AI tools may result in higher healthcare expenditures and less accessibility for the general public [30]. Since some patients might feel uncomfortable if their care is solely determined by computer-driven recommendations, it’s also crucial to take the doctor-patient relationship into account. Additionally, due to inadequate representation in the training data, language models may not have the necessary knowledge in cases involving uncommon pathologies or presentations [31,32]. As a result, healthcare professionals must be cautious when utilizing AI, use their clinical judgment, and interpret the language model’s recommendations in light of the particular circumstances surrounding each patient.

It is important to acknowledge both the strengths and weaknesses of AI in cardiology. By reducing the number of possible diagnoses and offering suggested treatments, language models like ChatGPT-4 can help medical professionals in diagnosing and treating patients [16]. Large patient data sets are processed efficiently and expertly by language models, allowing them to identify associations and provide insightful information. Healthcare providers can increase productivity and improve decision-making by making use of AI capabilities [33]. However, it is imperative to keep in mind that AI should not replace the role of healthcare professionals. In order to provide patients with the best care possible, clinical judgment and the human touch are still crucial. While language models are capable of data analysis, they might ignore the contextual factors that affect treatment choices. A balance must be struck between using AI as a tool to support clinical expertise and providing healthcare professionals with insightful information while ensuring that final decisions are made jointly by healthcare providers and patients. Maintaining trust and preserving the doctor-patient relationship are facilitated by open discussions about the role of AI in healthcare and transparency with patients, which ultimately results in safe, efficient, and individualized care.

Language models are powerful tools that have been trained using vast amounts of data to understand and generate human language. The quality and quantity of the data they are trained on, however, have a limit on how accurate and useful their predictions or recommendations can be. In the case of language models used in healthcare, the data used to train them is frequently restricted to electronic health records (EHRs) or medical literature, which may not accurately capture the entire scope of clinical practice or the complexity of individual patients. These restrictions could lead to bias in language models or a failure to consider all relevant factors that might influence a patient’s diagnosis or course of treatment. For example, language models may not have access to all relevant lab results, imaging studies, or other diagnostic tests, which can be critical in making an accurate diagnosis. Additionally, language models may not be able to consider the context of a patient’s individual circumstances, such as their medical history, lifestyle, or social determinants of health. Furthermore, language models may be particularly prone to inaccuracies in cases where patients have rare pathologies or unusual presentations. This is because these cases may not be well-represented in the training data, meaning that the language model may not have sufficient knowledge to make accurate predictions or suggestions. In such cases, healthcare providers must use their clinical judgment and expertise to evaluate the language model’s recommendations and adjust them accordingly.

The selection of appropriate training data is of utmost importance in generating optimal outputs. In the context of language models, using high-quality and diverse training data can significantly enhance the accuracy and relevance of the generated text. Making sure to use relevant and trustworthy medical data is essential for producing trustworthy and medically sound information when it comes to applications of language models in the medical field, like ChatGPT. The quality and relevance of the training data used have a significant impact on the accuracy and reliability of AI-generated insights. The use of comprehensive and high-quality medical data must be given top priority in the healthcare industry for patient safety. Data from EHRs, peer-reviewed medical journals, and only other reliable sources should be included. AI models like ChatGPT can produce more accurate and trustworthy information by utilizing substantial and comprehensive datasets, empowering healthcare professionals to make wise decisions and give patients the best care possible. The meticulous selection and curation of training data play a vital role in maintaining the integrity and trustworthiness of AI applications in the medical field. It is worth noting that ChatGPT has demonstrated impressive capabilities, including passing medical board exams, which highlights the potential of language models to assist and augment healthcare professionals [34].

It is essential to understand that language models are not a replacement for human healthcare providers. While they can be powerful tools to augment clinical decision-making, they should be used in conjunction with a provider’s clinical judgment and expertise. Ultimately, it is the responsibility of the healthcare provider to ensure that the care they provide is safe, effective, and personalized to the individual needs of each patient.

As AI and language models become more prevalent in healthcare, it is crucial that healthcare professionals receive adequate training to use these tools effectively. This training should cover not only the technical aspects of using AI but also the ethical and legal implications of its use. The healthcare professionals who use AI tools must understand the strengths and limitations of the technology, the importance of verifying its accuracy, and how to interpret the results generated by the tool. This training should also include discussions on the potential biases and limitations of AI and how these could impact patient care. Proper training is especially important because improper use of AI technology could have negative consequences for patient outcomes. For example, relying solely on a language model’s recommendation could lead to misdiagnosis or incorrect treatment decisions, potentially harming patients. Additionally, if the AI model is not properly trained or verified, it may produce inaccurate or misleading results that could lead to unintended consequences. Another potential risk is the impact of AI on the doctor-patient relationship. Patients may become concerned if they feel that their care is being determined solely by a machine, rather than by a human healthcare professional who can provide a personalized approach. It is essential to note that while AI can be a powerful tool in healthcare, it is not a replacement for healthcare professionals. The use of AI should be viewed as an augmentation of clinical decision-making rather than a replacement for it. Ultimately, the healthcare professional is responsible for the care and well-being of their patients, and they must use their clinical judgment to interpret the results generated by AI tools and provide individualized care to each patient. 

The major limitation of this study is that many of the ideas presented are hypothetical and reflect the perspectives of the authors. This is partly because language AI is still in its early stages in terms of its application to cardiology aspects. While ChatGPT-4, particularly when used with chatbots, is a valuable tool for human-machine conversations and generating meaningful text, its specific application to the field of cardiology may still require further development and validation.

## 4. Conclusions

In summary, language models have a range of uses and benefits in cardiology, including medical diagnosis and decision support, patient communication and education, and medical education and training. These benefits can improve patient outcomes, enhance medical education, and advance clinical research in the field of cardiology. While language models have these positives, there are also several pitfalls and limitations. These limitations should be carefully considered and addressed to ensure the safe and effective use of language models in cardiology.

## 5. Future Directions

Although language models like ChatGPT-4 have a lot of potential in the field of cardiology, a lot of work needs to be completed to create more precise and customized language models. Future research should focus on enhancing the quantity and quality of training data as well as adding more diverse types of data to language models, such as social determinants of health, wearable device data, and genomic data. This might aid in addressing some of the shortcomings and biases present in language models currently used in healthcare.

The incorporation of language models into clinical decision-making workflow is a crucial area for future research. This would entail creating intuitive dashboards and user interfaces that facilitate access to and interpretation of language model results by healthcare professionals. Further work needs to be conducted on creating more complex algorithms that can produce predictions or recommendations as well as explain their justification. This would promote transparency and trust in language models used in the healthcare industry.

Last but not least, future studies should focus on addressing some of the moral and legal issues that come up when using language models in healthcare. This might entail establishing uniform protocols for the application of language models in clinical settings as well as dealing with concerns about patient privacy and consent. Research also needs to be conducted on creating more thorough methods for assessing the security and effectiveness of language models in healthcare.

In conclusion, even though there are still many obstacles to be overcome, the application of language models in cardiology has the potential to completely alter how medical care is provided. With continued research and development, language models could help improve patient outcomes and enhance physician productivity. 

## Data Availability

Not applicable.
